# Clinical Aspects of Stevens-Johnson Syndrome/Toxic Epidermal Necrolysis With Severe Ocular Complications in India

**DOI:** 10.3389/fmed.2021.643955

**Published:** 2021-08-27

**Authors:** Swapna S. Shanbhag, Virender S. Sangwan, Aastha Singh, Pragnya R. Donthineni, Sayan Basu, Bhaskar Srinivasan, Shweta Agarwal, Geetha Iyer

**Affiliations:** ^1^The Cornea Institute, LV Prasad Eye Institute, Hyderabad, India; ^2^Department of Cornea, Dr. Shroff's Charity Eye Hospital, New Delhi, India; ^3^Center for Ocular Regeneration, LV Prasad Eye Institute, Hyderabad, India; ^4^Brien Holden Eye Research Centre, LV Prasad Eye Institute, Hyderabad, India; ^5^CJ Shah Cornea Services/Dr. G. Sitalakshmi Memorial Clinic for Ocular Surface Disorders, Medical Research Foundation, Sankara Nethralaya, Chennai, India

**Keywords:** Stevens-Johnson syndrome, toxic epidermal necrolysis, corneal blindness, limbal stem cell deficiency, lid margin keratinization, amniotic membrane, mucous membrane grafts

## Abstract

Stevens-Johnson syndrome/toxic epidermal necrolysis (SJS/TEN) is a spectrum of rare, severe immunological blistering skin reactions which are triggered by medication intake or infections. The acute phase is characterized by necrolysis of the skin and desquamation of mucosa, primarily oral and ocular, with significant mortality rates. The chronic phase is characterized by multi-organ sequelae with increased rates of morbidity and reduced quality of life for patients who have survived the acute phase. Since the primary goal in the acute phase is saving the life of the patient, ocular involvement is often missed and a significant proportion of patients present to an ophthalmologist with the chronic ocular sequelae. In India, chronic ocular sequelae and low vision are observed in two-thirds of patients who present in the chronic phase of SJS/TEN. In the chronic phase of ocular involvement, there are definite windows of opportunity which if targeted with specific interventions such as scleral lenses and mucous membrane grafts can help reduce the incidence of corneal blindness and improve the quality of life for patients with SJS/TEN. Over the last decade, several studies from India have advanced the understanding of the natural course of ocular involvement in SJS/TEN and the outcomes of timely interventions in the chronic phase of the disease. We present an overview of the epidemiology of ocular complications of SJS/TEN in India, the specific challenges faced in the management of ocular complications in the acute stage and recent advances in management of the chronic ocular complications of the disease.

## Introduction

Corneal blindness and ocular morbidity caused due to the ocular sequelae of SJS/TEN are challenging to address and treat. The ocular involvement of the disease is broadly categorized into an acute phase and a chronic phase. The acute phase usually constitutes the first 2 weeks from the onset of the disease. Each phase has its disease characteristics, presentation features, management options and preventive measures. In lieu of our recent understanding related to several of these distinct parameters and their pathophysiology, there has been a paradigm shift in management strategies in each phase.

The exact difference in incidence and prevalence of the disease and the severity of its ocular complications/sequelae among various parts of the world is not known. Based on published reports, this seems to be high in India. Few of the largest series of chronic ocular sequelae of SJS and their management are from India highlighting this possibility.

The goal of this review article is therefore to present the Indian perspective of the disease magnitude, clinical features, current management options including recent and ongoing research, and future directives. In addition, challenges unique and specific to the Indian subcontinent, existing lacunae and possible means to overcome these in the future will be highlighted.

## Disease Magnitude/Epidemiology of SJS/TEN in India

The annual incidence of SJS/TEN in India is not known. A systematic review on cutaneous adverse drug reactions (CADRs) in the Indian population calculated an incidence rate of 9.22/1000 cases, out of which SJS/TEN were the most common severe CADRs (6.84%) (1). In a study by Sushma et al., 19.5% of hospitalized patients with severe cutaneous adverse reaction (SCAR) over 9 years were diagnosed with SJS/TEN (2). Although a rare condition, the overall mortality of SJS/TEN in the Indian population is estimated to be 12.94% with 3.92% in SJS cases, 5.26% in SJS-TEN overlap cases and 28.20% in TEN cases, thus signifying the gravity of this disease (3).

Based on published literature from India, the most common etiology for SJS/TEN was drug intake (97.14%) (3). The most common culprit drugs were antibiotics (37.27%), anti-epileptics (35.73%), and non-steroidal anti-inflammatory drugs (NSAIDs - 15.93%) (3). Individual drugs that were identified were carbamazepine (18.25%), phenytoin (13.37%), fluoroquinolones (8.48%), paracetamol (6.17%), and sulfonamides (6.16%). Regional differences were observed, with South Indian studies reporting a higher percentage of cases with the causative factor being fluoroquinolones, while West and North Indian studies reporting a higher percentage of cases with the causative factor being sulfonamides (3).

A systematic review reported that 40.29% patients of acute SJS/TEN in the Indian population had ocular complications in the acute phase, making this one of the most common organ systems affected in the acute phase (3). For the patients who suffer chronic ocular sequelae, the most identified etiology was ingestion of a drug (58–78%), the most common of which were sulfonamide antibiotics (49%), mainly cotrimoxazole (30%) followed by NSAIDs and antiepileptic drugs (4–7).

The largest published studies from India on chronic ocular complications related to SJS/TEN reported that the mean duration from onset of SJS/TEN to presentation to a corneal specialist is ~3.8–7 years (6–10), with 41–66% patients presenting more than a year after acute SJS/TEN (6, 7). Sixty percent eyes of patients in the chronic phase presented with low vision or blindness (6). Lid abnormalities were observed in 97% eyes, conjunctival complications in 65% eyes, and corneal complications in 85% eyes (6). In a study from India that evaluated pediatric patients with dry eye, 33% eyes were observed to have the etiology of SJS/TEN and 38% of these eyes had severe visual impairment or blindness (11). Another study observed that 23% patients who presented to a tertiary ophthalmological institute with bilateral limbal stem cell deficiency (LSCD) in India had the etiology of SJS/TEN (12).

## Clinical Features and Current Management Options

### Acute Phase

Recent published studies have shown that amniotic membrane transplantation (AMT) in the acute phase significantly reduces the incidence of severe chronic ocular complications and subsequent corneal blindness. A randomized controlled trial performed in India which compared the outcomes of AMT with conventional medical therapy in the form of topical steroids, topical antibiotics, topical lubricants clearly showed that AMT in combination with medical therapy prevented chronic ocular sequelae at a follow-up of 6 months (13). In patients that underwent AMT, not a single eye suffered from corneal haze, LSCD, symblepharon, ankyloblepharon, or lid-related complications. However, almost 99% of patients presenting to ophthalmological institutions in the chronic phase after a period of a year or more of acute SJS/TEN had not undergone AMT in the acute phase in India (6, 7).

### Chronic Phase

Reduced attention to ocular care in the acute stage either due to lack of accessibility to an ophthalmologist or of awareness has led to a high incidence of chronic ocular complications. Many studies have been published from India on different aspects of the chronic stage sequelae. These study inferences have been highlighted in this section.

#### Diagnosis of SJS/TEN in the Chronic Phase of Advanced Cicatricial Conjunctivitis

In India, SJS/TEN is the referral diagnosis for most patients with advanced chronic cicatricial conjunctivitis (CCC). Detailed documentation of the drug history and the acute phase of SJS/TEN may not be provided by all patients. Shanbhag et al. recently published a scoring system to ensure correct diagnosis of SJS/TEN in patients who present with advanced CCC (14). Vazirani et al. have used this scoring system in an algorithm to reach a specific diagnosis in eyes with CCC thus helping in appropriate decision-making regarding further medical and surgical interventions in these eyes (15).

#### Classification of Chronic Sequelae

A classification or a scoring system for involvement of different parts of the ocular surface in these eyes provides a quantitative tool to identify progression of disease and to compare outcomes of various medical and surgical interventions, especially in the chronic phase of SJS/TEN. A scoring system has been proposed by Sharma et al. to reflect the advanced severity of the chronic ocular surface complications that are commonly seen in India in patients with SJS/TEN (16). Sharma et al. scored 12 ocular surface parameters (6 corneal, 3 conjunctival, and 3 eyelid) ranging from 0 to 5 and demonstrated that there was a co-relation with corrected distance visual acuity.

#### Ocular Surface Flora and Incidence of Microbial Keratitis in the Chronic Phase

Venugopal et al. prospectively evaluated the conjunctival flora in Indian patients with SJS/TEN and noted that 59% eyes in the SJS/TEN group had positive bacterial cultures from conjunctival swabs as compared to 13% eyes in patients without SJS/TEN (17). The most common isolate was Corynebacteria species in 34% eyes followed by coagulase-negative staphylococci in 29% eyes. In eyes with SJS/TEN that developed microbial keratitis, Bagga et al. noted positive microbiological cultures in 69% eyes with isolated bacterial infections in 60% eyes and polymicrobial infections noted in 38% eyes (18). The most common bacteria isolated were Staphylococcus species (35% eyes). Sharma et al. prospectively studied eyes with microbial keratitis and noted isolated bacterial infections in 63% eyes with polymicrobial infections in 29% eyes (19). Corneal perforation was noted in 31% eyes out of which 70% of eyes required a therapeutic penetrating keratoplasty, thus demonstrating a higher need for surgical interventions in these eyes.

#### Lacrimal Gland in the Chronic Phase of SJS/TEN

Recent studies from India by Singh et al. have clearly shown that in eyes with severe SJS/TEN with no secretion of aqueous component of tears, the morphology of the acinar structures of the orbital lobe is normal thus establishing the fact that dry eye in SJS/TEN is not secondary to primary involvement of the lacrimal gland (20, 21). This gives more credence to the theory that fibrosis of the superotemporal conjunctiva around the ductules of the lacrimal gland which drain tears onto the ocular surface is the main etiology for aqueous deficiency dry eye in patients with SJS/TEN. The morphological appearance of the lacrimal gland has also been described by Singh et al. in patients with SJS where the gland appears to have a flat contour with subepithelial scarring, sometimes accompanied by symblepharon in the region of the lacrimal gland with engorged conjunctival vessels (22). Singh et al. also described that the amount of ductules actively secreting tears were significantly reduced or absent in eyes with SJS/TEN as compared to normal eyes (23).

#### Treatment Options

##### Lid Margin Keratinization

The exact etiopathogenesis for LMK is not yet known, however various pathophysiological mechanisms have been put forth by Singh et al. in a recently published study that described the histopathological features of excised keratinized lid margins (24).

**Figure 1:**
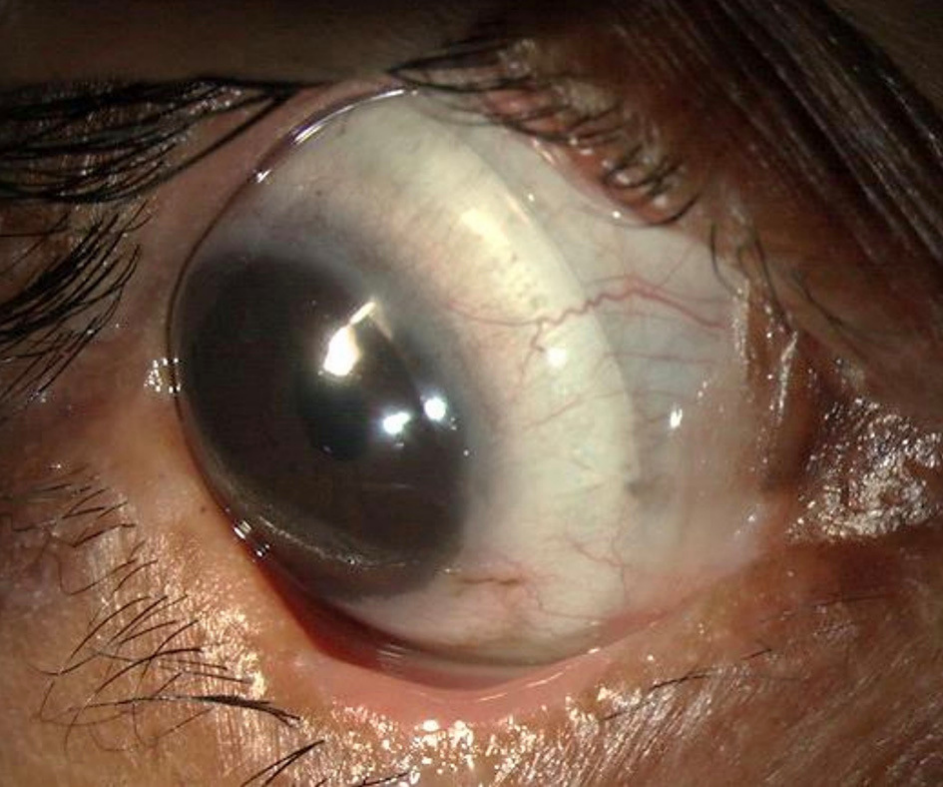
Scleral lenses [prosthetic replacement of ocular surface ecosystem (PROSE) lenses] used for management of severe dry eye in a patient with chronic ocular sequelae post Stevens-Johnson syndrome/toxic epidermal necrolysis.

**Figure 2:**
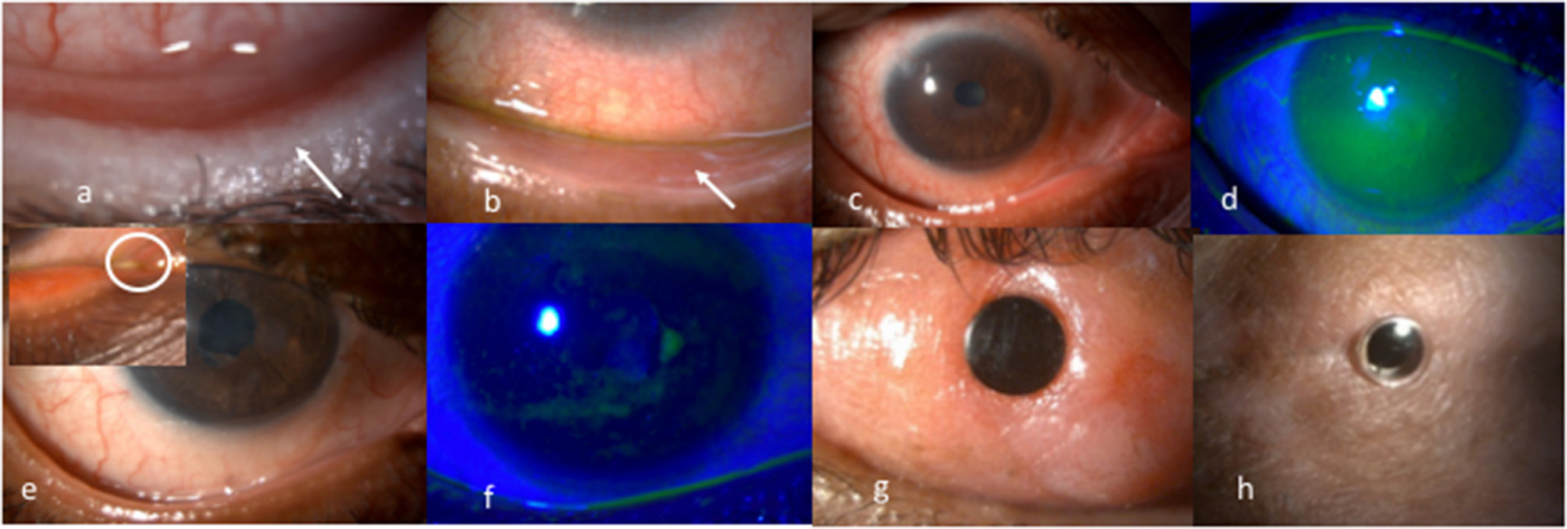
**(a)** Lower lid margin keratinization causing a sand paper effect on the ocular surface due to blink related microtrauma. **(b)** Post mucous membrane grafting for lid margin keratinization showing a smooth lid margin maintained at years following surgery. **(c,d)** Inflamed ocular surface with diffuse superficial punctate keratopathy on corneal fluorescein staining pre punctal cautery. **(e,f)** Resolution of surface inflammation and punctate keratopathy post punctal cautery improving patient's symptoms of photophobia and foreign body sensation. **(g)** 15 years post modified osteo odonto keratoprosthesis maintaining a best corrected visual acuity of 20/20. **(h)** 5 years post Boston type 2 keratoprosthesis maintaining best achieved corrected visual acuity of 20/30.

Most corneal complications in SJS/TEN can be attributed to LMK. For the treatment of the lid-related keratopathy (LRK) secondary to LMK, two modalities that have been most effective are prosthetic replacement of ocular surface ecosystem (PROSE) lenses and lid margin mucous membrane grafts (MMG). In addition to preventing keratopathy, PROSE lenses help reduce symptoms due to dry eye and improve vision in patients with corneal scarring (Figure 1). MMG harvested from the oral mucosa after excision of keratinized lid margin helps prevent keratopathy (Figures 2a,b). The surgical technique for lid margin MMG has been published recently (25).

The first study from India to evaluate the outcomes of MMG in eyes with LRK due to SJS/TEN was published by Iyer et al. in 2010 (8). In this study, 93% eyes had an improved ocular surface and improved BCVA (best corrected visual acuity) over 6 months of follow-up and patients demonstrated significant improvement in comfort. In another retrospective largest interventional study in SJS over 25 years by Iyer et al.; symptomatic improvement was noted in 88% with recurrence of keratinization in 8.4% (10). MMG was found to have a beneficial effect on the ocular surface by reducing the corneal vascularization, haze and punctate keratopathy, thereby aiding in improved vision. A well-performed surgery is a key to a beneficial outcome corroborated by tear cytokine analysis (26). In addition, the role of an altered retinoid metabolism in etiopathogenesis as well as the impact of mucous membrane grafting on it was studied. A corelation pointing toward a possible therapeutic role of topical retinoic acid in a select subgroup of patients was noted (27). Further studies are required to clearly understand the exact role of retinoid metabolism and ocular sequelae of SJS.

Subsequent studies from India by Basu et al. and Shanbhag et al. studied the natural history of LRK due to LMK and noted that definitive therapy in the form of MMG and PROSE lenses significantly improve BCVA and prevent development or progression of LRK when compared to conservative therapy in the form of topical medications (6, 7). A combination of MMG and PROSE lenses helped in maintaining and improving BCVA in eyes with LRK, with better outcomes in the pediatric age group with MMG while PROSE lenses showed better outcomes in the adult age group while a combination of both showed the best outcomes in both groups regardless of age (7).

##### Dry Eye

Iyer et al. reported an improvement in 45.8% and stability of surface in 53.6% of the 231 eyes where punctal cautery was done in chronic SJS (10) (Figures 2c–f). Outcomes of minor salivary gland transplantation in eyes with SJS/TEN performed in Indian patients have also been encouraging (28).

##### Fornix Reconstruction and Adnexal Procedures

**Figure 3:**
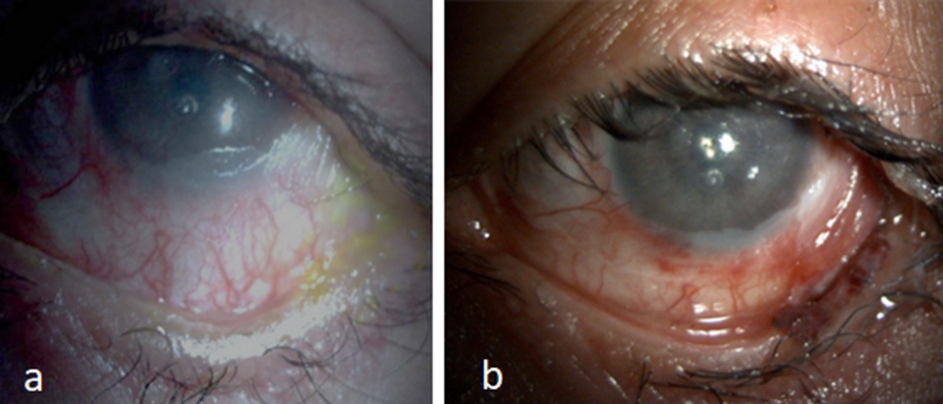
**(a)** Keratinized surface with shortened fornix. **(b)** Two years following fornix reconstruction with mucous membrane and amniotic membrane graft with relatively moist surface and improvement in corneal luster using PROSE lens.

Fornix reconstruction and symblepharon release using an amniotic membrane (AM), MMG or with COMET (cultivated oral mucosal epithelial transplantation) help address unstable tear film, exposure keratopathy and improved fitting of PROSE lenses in eyes with shortened fornices (9, 10, 29, 30) (Figures 3a,b). Outcomes of COMET in eyes with SJS/TEN have been described from two centers in India with both showing improved outcomes and restoration of the anatomy of the ocular surface (31, 32). Successful resolution of recurrent cicatricial entropion due to SJS/TEN was shown by Singh et al. with the use of labial mucosa for spacing the anterior lamella and reconstruction of the lid margin and posterior lamella with minimal recurrence rate at a follow-up period of 16 months (33).

##### Cataract Surgery in SJS/TEN

Decrease in vision due to cataract or localized corneal opacity can be addressed with cataract extraction and/or an optical iridectomy, respectively. Cataract surgery in SJS/TEN can be challenging in the setting of a poor ocular surface, severe dry eye, corneal scar, and symblepharon. Surface stabilization procedures should be performed before undertaking cataract surgery. Three studies from India have described the outcomes of cataract surgery in patients with SJS/TEN (34–36). Phacoemulsification was the most common surgical procedure performed. Measures such as endo-illuminator use for better visualization and use of viscoelastic on the corneal surface throughout the surgery have been described to improve the outcomes in these eyes.

##### Keratoprosthesis in SJS/TEN

Keratoprosthesis (kpro) forms the mainstay of treatment in the end-stage of the disease (37) (Figures 2g,h). Given the high risk for complications, the Boston Type 1 Kpro is not routinely recommended in moist SJS eyes. The outcomes of different kpro devices in eyes with SJS/TEN such as the modified osteo-odonto keratoprosthesis, the Boston Type 2 Keratoprosthesis, the LVP keratoprosthesis, and the Lux keratoprosthesis have been published from India (38–42). Although the incidence of complications are higher in eyes with SJS/TEN, the outcomes with all the keratoprostheses devices have been shown to be encouraging in patients with end-stage corneal blindness. In the authors' experience, the outcome of any keratoprosthesis is best when it is performed in an eye that has not undergone prior multiple keratoplasties.

## Specific Challenges

Lack of a registry or database of SJS affected individuals in the country makes it difficult to estimate the incidence and prevalence of the disease. Routine use of over the counter medications and common use of sulphonamide antibiotics for infections in primary health centers could be a factor responsible for increased occurrence of the condition. General practice of deferring seeking immediate medical care in India for a disease condition that simulates chicken pox in the early stage delays presentation to a health care facility. This practice shortens the time in the acute phase to offer treatment and delays initiation of ophthalmic care in the short window period available. Lack of accessibility to an ophthalmologist in a center that provides primary intensive care coupled with the lack of awareness of the role of an ophthalmologist in the acute phase further compromises the ocular care in the acute phase. Furthermore, immediate referral, post the acute phase, to an ophthalmologist to monitor the onset and course of chronic sequelae is not a routinely adopted practice. This further delays the identification and implementation of preventive or treatment strategies early on. Similarly, the delay in referral to a cornea specialist till the occurrence of a manifest significant decrease in visual acuity, by which time the ocular surface is irreversibly affected, closes the doors on availing the windows of opportunities.

## Ongoing Research and Future Directives

### Promoting Awareness

The role of an ophthalmologist in the acute phase needs to be emphasized. In addition, the need for and the benefits of AMT in the acute phase, where indicated, has to be impressed upon ophthalmologists. This would help promote bedside consultations from an ophthalmologist as soon as the disease is diagnosed. Likewise, the need for early referral in the chronic phase to cornea specialists, irrespective of visual acuity, has to be asserted. This process of creating and spreading awareness has begun and will continue to gain momentum. India has been in the forefront in advancing research and care in this field through several recently published works of importance. This has helped create a spotlight for the condition amongst ophthalmologists in the country contributing to improved awareness. Additional measures to promote awareness could include interdisciplinary meetings among ophthalmologists, intensivists, pediatricians and dermatologists, alongside specialty fellowships.

### Genotyping for Prevention of SJS/TEN

The most sought approach to prevention of SJS would be preemptive HLA (human leukocyte antigen) genotyping before prescription of a drug. Strong associations between drugs and HLA types have been studied, thus making an individual high-risk to develop SJS/TEN if they possess a certain HLA type, and they were to ingest a specific drug (43, 44). If preemptive genotyping is performed to screen the HLA type before prescribing the medication, it may be possible to prevent SJS/TEN. There are studies which have performed HLA genotyping in patients who have developed SJS/TEN to carbamazepine, phenytoin, lamotrigine, levetiracetam, and cold medications in the Indian population (45–53). Significant associations have been noted between SJS/TEN secondary to carbamazepine intake and HLA-B*15:02, HLA-A*31:01, HLA-B*57:01, and HLA-DRB1*07:01 (45, 46, 50, 54). Also, significant associations have been noted between HLA-A*33:03, HLA-B*44:03, and HLA-C*07:01 and patients with SJS/TEN and severe ocular complications secondary to cold medicine (multi-ingredient cold medications and NSAIDs) intake (51, 52). A significant genome-wide association has been noted between SJS/TEN secondary to cold-medicine intake and *IKZF1* SNPs (single nucleotide polymorphisms) in the Indian population with severe mucosal involvement suggesting that IKZF1 might be a potential marker for cold-medicine related SJS/TEN (55). To make preemptive genotyping effective, multicentre studies analyzing drug-HLA associations are required to study the cost-effectiveness. Pre-emptive genotyping for known associations would be the way forward facilitated by curtailed costs to conduct the same.

### Monitoring

A national database to monitor adverse drug reactions could advance our knowledge of the exact drugs and the components in these drugs that are responsible for causing SJS/TEN. A registry-based approach to document all cases of SJS/TEN nationally could help in projecting the true incidence rates.

### Further Research

The burden of ocular morbidity in the country due to SJS/TEN is huge with most patients presenting with severe ocular surface disorders that need surgical interventions. Several issues related to etiopathogenesis of specific organ/tissue damage in SJS, including lacrimal gland and lid margin remains yet to be explored and understood. This would help further potential therapeutic strategies.

## Conclusion

The cumulative data from the Indian subcontinent in the field of ocular sequelae of SJS, clinical as well as research oriented, is substantial. Nevertheless, the task ahead to better define several parameters as well as refine our understanding of etiopathogenesis specific to the country, if any, and therefore develop appropriate management strategies, is huge. The authors believe that further studies in the above said directions will help pave the way forward.

## Author Contributions

SS, VS, AS, PD, SB, BS, SA, and GI: concept and design of the study, drafting the article or revising it critically for important intellectual content, and final approval of the version to be published. SS, AS, and GI: literature search. All authors contributed to the article and approved the submitted version.

## Conflict of Interest

The authors declare that the research was conducted in the absence of any commercial or financial relationships that could be construed as a potential conflict of interest.

## Publisher's Note

All claims expressed in this article are solely those of the authors and do not necessarily represent those of their affiliated organizations, or those of the publisher, the editors and the reviewers. Any product that may be evaluated in this article, or claim that may be made by its manufacturer, is not guaranteed or endorsed by the publisher.
